# Importance of diphthamide modified EF2 for translational accuracy and competitive cell growth in yeast

**DOI:** 10.1371/journal.pone.0205870

**Published:** 2018-10-18

**Authors:** Harmen Hawer, Koray Ütkür, Meike Arend, Klaus Mayer, Lorenz Adrian, Ulrich Brinkmann, Raffael Schaffrath

**Affiliations:** 1 Institut für Biologie, Fachgebiet Mikrobiologie, Universität Kassel, Kassel, Germany; 2 Roche Pharma Research & Early Development, Large Molecule Research, Roche Innovation Center München, Penzberg, Germany; 3 AG Geobiochemie, Department Isotopenbiogeochemie, Helmholtz-Zentrum für Umweltforschung GmbH–UFZ, Leipzig, Germany; 4 Fachgebiet Geobiotechnologie, Technische Universität Berlin, Berlin, Germany; John Curtin School of Medical Research, AUSTRALIA

## Abstract

In eukaryotes, the modification of an invariant histidine (His-699 in yeast) residue in translation elongation factor 2 (EF2) with diphthamide involves a conserved pathway encoded by the *DPH1*-*DPH7* gene network. Diphthamide is the target for diphtheria toxin and related lethal ADP ribosylases, which collectively kill cells by inactivating the essential translocase function of EF2 during mRNA translation and protein biosynthesis. Although this notion emphasizes the pathological importance of diphthamide, precisely why cells including our own require EF2 to carry it, is unclear. Mining the synthetic genetic array (SGA) landscape from the budding yeast *Saccharomyces cerevisiae* has revealed negative interactions between EF2 (*EFT1*-*EFT2*) and diphthamide (*DPH1*-*DPH7*) gene deletions. In line with these correlations, we confirm in here that loss of diphthamide modification (*dph*Δ) on EF2 combined with EF2 undersupply (*eft2*Δ) causes synthetic growth phenotypes in the composite mutant (*dph*Δ *eft2*Δ). These reflect negative interference with cell performance under standard as well as thermal and/or chemical stress conditions, cell growth rates and doubling times, competitive fitness, cell viability in the presence of TOR inhibitors (rapamycin, caffeine) and translation indicator drugs (hygromycin, anisomycin). Together with significantly suppressed tolerance towards EF2 inhibition by cytotoxic *DPH5* overexpression and increased ribosomal -1 frame-shift errors in mutants lacking modifiable pools of EF2 (*dph*Δ, *dph*Δ *eft2*Δ), our data indicate that diphthamide is important for the fidelity of the EF2 translocation function during mRNA translation.

## Introduction

The conversion of a histidine residue to diphthamide is a posttranslational modification unique to translation elongation factor 2 (EF2) [1]. The name refers to the target role diphthamide modified EF2 plays for diphtheria toxin and other lethal ADP ribosylases [2,3]. As a result, diphthamide dependent ADP ribosylation by bacterial toxins inactivates the essential translocase function of EF2 in mRNA translation, blocks protein synthesis and eventually leads to cell death [4,5]. Similarly, diphthamide mediates killing of fungi including the budding yeast *Saccharomyces cerevisiae* by sordarin [6,7], an antibiotic which irreversibly locks the modified EF2 on the ribosome [8]. So, diphthamide clearly represents an *Achilles heel* on EF2 with pathological relevance for cell growth and proliferation control [9–11]. Its physiological role, however, is less clear although diphthamide can be found on archaeal and eukaryal EF2 [10,11].

**Fig 1:**
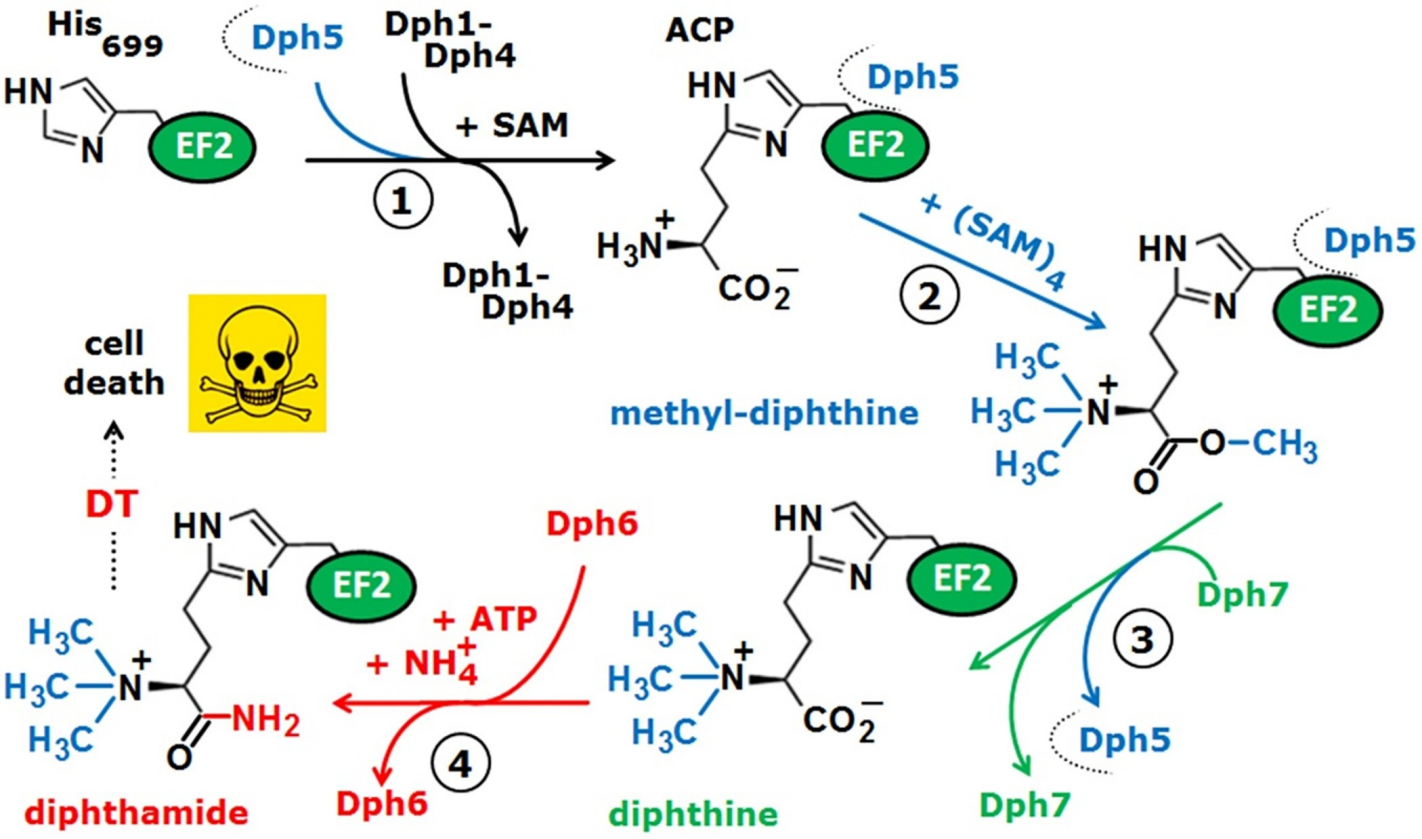
Diphthamide synthesis on yeast EF2 involves a multi-step pathway. In step one (**1**, black label), Dph1-Dph4 use SAM as amino-carboxyl-propyl (ACP) donor to modify His-699 in EF2 with ACP. Next (**2**, blue label), Dph5 generates methyl-diphthine using the methyl donor function of SAM. Subsequently (**3**, green label), this intermediate is converted to diphthine by demethylase Dph7 before finally (**4**, red label), Dph6 generates diphthamide from diphthine using ammonium and ATP. Diphthamide can be ADP-ribosylated by diphtheria toxin (DT) to inactivate EF2 and cause cell death. Note that prior to ACP formation, Dph5 binds to unmodified EF2 and dissociates from intermediate methyl-diphthine in a fashion licensed by Dph7 [19,50]. The model is derived and up-dated from Schaffrath *et al*. (2014) [11].

Indeed, its formation is conserved from archaea to yeast and man and in eukaryotes, involves a multi-step biosynthetic pathway (Fig 1) that is encoded by the DPH1-DPH7 gene network [12–19]. Starting with the addition of a 3-amino-3-carboxypropyl (ACP) radical from S-adenosyl-methionine (SAM) to the imidazole ring of the crucial histidine in EF2 (yeast: His-699; humans: His-715), step one of the pathway (Fig 1) engages Fe/S enzyme (Dph1•Dph2) chemistry, electron transfer (Dph3) and a putative J-type chaperone (Dph4) to form ACP-modified EF2 [20–27]. Multiple methylation of this first intermediate by a methylase (Dph5) generates methyl-diphthine [28–31] (Fig 1). Next, the latter is converted to diphthine by a demethylase (Dph7) before an amidase (Dph6) with the use of ATP and ammonium generates the end product diphthamide [17,18,31] (Fig 1). Remarkably, Dph5 binds to unmodified EF2 prior to ACP formation, and its dissociation from methyl-diphthine depends on Dph7 (Fig 1) [19]. Consistently, when diphthamide synthesis is incomplete (*dph7*Δ) or absent (*dph1*Δ *-dph4*Δ), excess Dph5 has been shown to bind EF2 [13,19,24] and in tandem with *DPH5* overexpression is cytotoxic due to EF2 inhibition [13,19,24]. Thus, apart from its catalytic activity as a methylase, Dph5 can negatively interfere with EF2 function in the absence of stepwise diphthamide synthesis on EF2 [13,19,24].

Despite the high degree of conservation in diphthamide synthesis, most yeast *dph* mutants are viable and grow normally under standard conditions [6,7,32]. In multicellular organisms, however, loss of diphthamide is pleiotropic and has been reported to pre-activate cell death receptor pathways [33] and associate with severe defects or diseases including ovarian and colorectal cancers [34–36], embryonic lethality, neuronal underdevelopment, intellectual disability, polydactyly and cranofacial abnormalities [26,27,37–39]. Clearly, while this emphasizes biomedical importance, it is unknown whether and how diphthamide defects in the above syndromes link up to the biological function of EF2 in mRNA translation. Based on ribosomal frame-shift assays in yeast, diphthamide has been proposed to support the fidelity of translocation by EF2 during mRNA translation [19,32,40]. How this is accomplished, is not entirely clear, and only recently have cryo-EM studies emerged that relate diphthamide modified EF2 in complex with the ribosomal decoding center to accurate mRNA translation [41,42].

To further study the function of diphthamide for EF2, we mined the yeast synthetic genetic array (SGA) database (TheCellMap.org) [43] and extracted *EFT1-EFT2* and *DPH1-DPH7* gene correlation profiles for experimental validation of genetic interaction *in vivo*. We find that the diphthamide modification becomes crucial for yeast cell performance upon EF2 down-regulation (*eft2*Δ). Thus, diphthamide loss together with EF2 undersupply (*dph*Δ *eft2*Δ) cause growth phenotypes induced by stressors including translation indicator drugs and culminate in competitive fitness defects. Furthermore, irrespective of cellular EF2 levels, diphthamide defects (*dph*Δ, *dph*Δ *eft2*Δ) trigger -1 frame-shift errors supporting the view that the modification is physiologically important for translational accuracy and EF2 function.

## Results

### SGA analysis reveals EF2 and diphthamide gene network correlations

Using high-density arrays of double mutants for systematic mapping of genetic interactions in yeast has enabled global SGA analysis of gene deletion collections [44]. For a given gene, the approach provides a genetic interaction landscape thereby generating phenotypic signatures diagnostic for functions of known genes or unassigned, cryptic ORFs [44]. Consistent with this notion, genes with similar interaction profiles often are functionally related in shared biochemical pathways or protein complexes [45,46].

**Fig 2:**
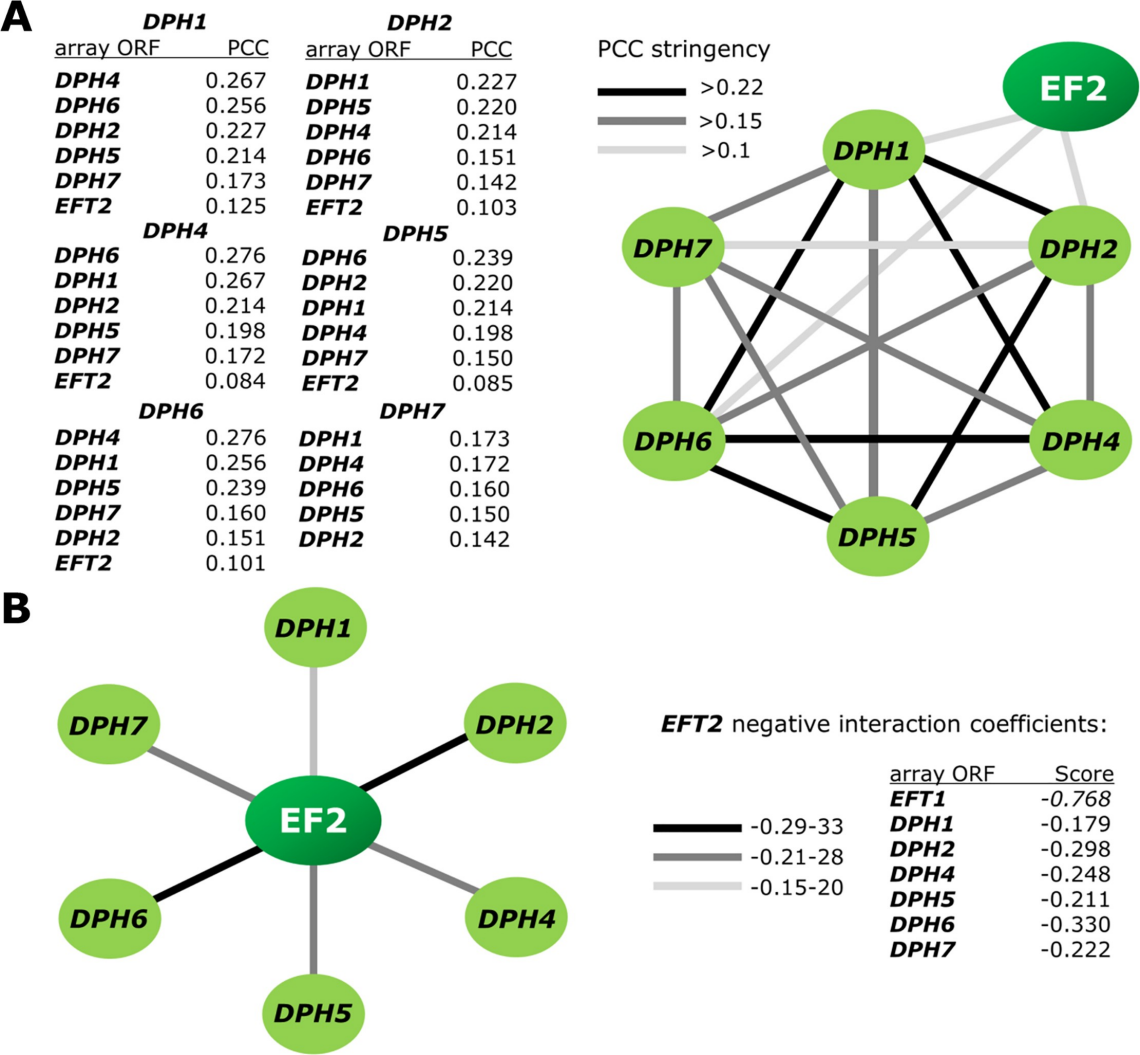
SGA analysis reveals strong correlation between EF2 and diphthamide gene networks. (A) Based on Pearson correlation coefficients (PCC) of indicated stringencies, each diphthamide query gene (*DPH1-DPH7*) correlates to other *DPH* gene members and to a lesser yet significant degree to EF2 (*EFT2*) in a tightly clustered interaction network. (B) SGA based coefficients of query gene *EFT2* identify strongest negative relations with *EFT1* and weaker albeit significant ones with diphthamide network genes (*DPH1-DPH7*). All SGA data were retrieved from TheCellMap.org database [43] (S1 File).

Therefore, we mined the SGA database update (TheCellMap.org) [43] to examine correlations between the interaction landscapes of EF2 (*EFT1-EFT2*) and diphthamide (*DPH1-DPH7*) genes (S1 File). We compared *EFT1*, *EFT2* and *DPH1-DPH7* query gene interactions with every array ORF in the database (S1 File) and ranked the similarity between all possible pairwise profiles according to their Pearson correlation coefficient (PCC) stringencies (Fig 2A). The survey revealed that with the exception of *DPH3/KTI11*, for which there are no entries in TheCellMap.org [43], all other *DPH* genes and *EFT2* scored significantly highly among the correlation profiles generated, placing them within a tightly clustered interaction network (Fig 2A). Here, *DPH1*, *DPH2* and *DPH6* and to a lesser degree *DPH4* and *DPH5* were found to correlate with *EFT2* (Fig 2A). Based upon a maximum score of negative genetic interaction (-0.768), the two EF2 gene paralogs (*EFT1* and *EFT2*) most strongly relate to one another and to a lesser, yet significant degree to the *DPH* genes (Fig 2B). Thus, TheCellMap.org [43] provides robust signatures indicating that the EF2 and diphthamide encoding networks correlate genetically and may functionally impact on each other.

### Loss of diphthamide and EF2 undersupply confer synthetic growth phenotypes

In order to validate the SGA correlations above, we tested the functional importance of EF2 and diphthamide interactions *in vivo* using *S*. *cerevisiae* as a model system. We compared growth of wild-type cells with that of mutants lacking (i) diphthamide biosynthesis (*dph2*Δ), (ii) one of the two EF2 gene copies (*eft2*Δ) or (iii) both (*dph2*Δ *eft2*Δ) under standard laboratory conditions and in response to thermal or chemical stressors.

**Fig 3:**
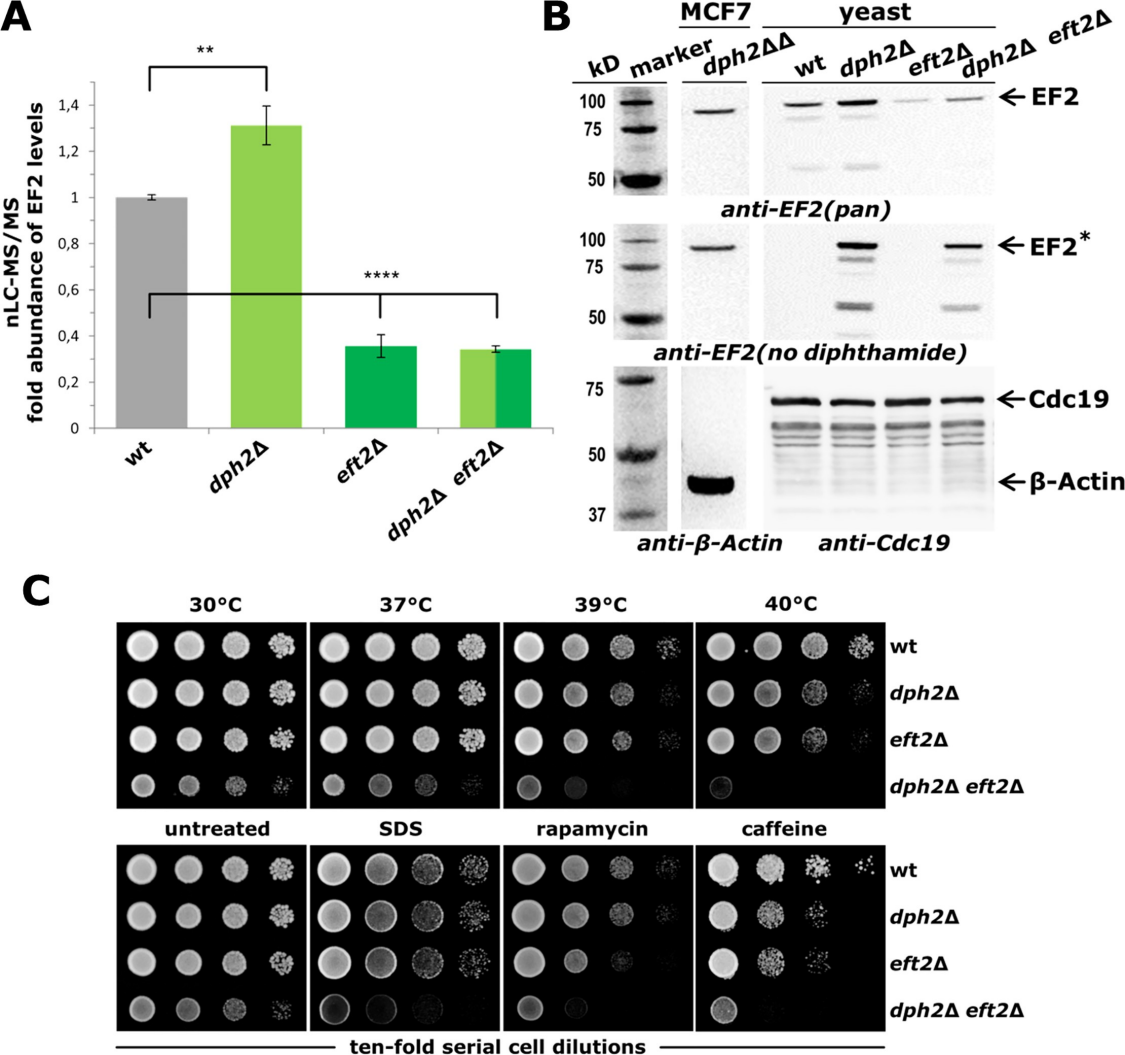
Loss of diphthamide modification and EF2 downregulation lead to synthetic growth phenotypes. (A) MS based quantification of EF2 levels from yeast mutants (*dph2*Δ, *eft2*Δ, *dph2*Δ *eft2*Δ) in relation to wild-type (wt) EF2 values (1.0). ** (P ≤ 0.01); **** (P ≤ 0.0001). (B) Western blots using antibodies for global EF2 recognition (*anti-EF2(pan)*, top panel), specific detection of EF2 pools that lack the diphthamide modification (*anti-EF2(no diphthamide)*, middle panel) and for internal protein expression controls (*anti-β-Actin* and *anti-Cdc19*, bottom panels). Protein extracts from homozygous (*dph2*ΔΔ) diphthamide-minus MCF7 [33] cell lines served as internal controls (all panels). (C) Synthetic sick growth phenotypes result from combining EF2 downregulation with loss of diphthamide. Ten-fold serial cell dilutions of yeast strains (as in A & B) were cultivated in the absence (untreated) or presence of various chemical stressors (SDS [0.2% w/v], rapamycin [15 nM] or caffeine [7.5 mM]) or at different temperatures (30°C, 37°C, 39°C or 40°C).

Relative quantification of EF2 expression levels by mass spectrometry (MS) showed that deleting one of the two EF2 genes (*eft2*Δ, *dph2*Δ *eft2*Δ) reduced EF2 amounts to about ~35% compared to wild-type levels (Fig 3A). The MS data were corroborated by detection of reduced EF2 levels in Western blots using antibody *anti-EF2(pan)* that was raised against human EF2 [33] and recognizes the counterpart from yeast (Fig 3B). The data imply that *EFT1*, which becomes essential for *eft2*Δ cell viability [47], plays a minor role in EF2 supply compared to *EFT2*. Notably, as judged from MS data and our *anti-EF2(pan)* Western blots, the diphthamide mutant (*dph2*Δ) maintaining proper EF2 gene dosage (*EFT1*, *EFT2*) produced higher-than-normal levels of EF2 (~1.3 fold) (Fig 3A and 3B). This is in striking contrast to the *dph2*Δ *eft2*Δ mutant and suggests that EF2 levels can be upregulated in response to lack of diphthamide synthesis provided both EF2 genes (*EFT1*, *EFT2*) are present.

In addition, we validated diphthamide modification states of EF2 using antibody *anti-EF2(no diphthamide)* recently reported to exclusively detect human EF2 that lacks the modification [33] (Fig 3B). Our Western blots confirm the cross-specificity of the antibody which allows for specific detection of unmodified yeast EF2 in diphthamide mutants (*dph2*Δ, *dph2*Δ *eft2*Δ) (Fig 3B). Moreover, the lack of *anti-EF2(no diphthamide)* responsive signals from diphthamide proficient (wild-type, *eft2*Δ) strains (Fig 3B) suggests that EF2 appears to be quantitatively modified in yeast, a scenario similar to near complete diphthamide modification of human EF2 [33]. Hence, use of *anti-EF2(no diphthamide)* in Western blots provides a facile and alternative means to detect and quantify yeast EF2 modification states.

Phenotypic assays with these verified strains demonstrate negative synthetic interactions and additive growth defects under standard laboratory and stress conditions. In particular, the composite mutant (*dph2*Δ *eft2*Δ) was significantly reduced in growth at elevated cultivation temperatures (39°C, 40°C) and in the presence of two TOR inhibitor drugs [48,49], rapamycin (15 nM) or caffeine (7.5 mM), as well as SDS (0.02% [w/v]) (Fig 3C). This is in contrast to the response of the single mutants (*dph2*Δ, *eft2*Δ) cultivated under thermal or chemical stress conditions, which perform very similar to the wild-type (Fig 3C).

To sum up, synthetic sick growth phenotypes as a result of combined EF2 (*eft2*Δ) and diphthamide (*dph2*Δ) gene deletions go hand-in-hand with the interaction landscapes predicted from the SGA data (S1 File; Fig 2) revealing that upon EF2 undersupply, yeast cell growth can indeed become dependent on the diphthamide modification (Fig 3). Its requirement for proper EF2 function is furthermore evident from an independent genetic scenario, in which we halved the EF2 gene complement in a *DPH4* deletion strain (*dph4*Δ *eft2*Δ) lacking Dph4, a J-type chaperone [26,27] cooperating with Dph2 in first step of diphthamide synthesis (Fig 1). The resultant double mutant (*dph4*Δ *eft2*Δ) copies the synthetic sick phenotypes (S1 Fig) of our reference mutant (*dph2*Δ *eft2*Δ) above (Fig 3C) thus supporting the SGA based predictions (Fig 2) and reinforcing that the EF2 (*EFT1-EFT2*) and diphthamide (*DPH1-DPH7*) gene networks are functionally correlated.

### Loss of diphthamide and EF2 undersupply interfere with competitive fitness

Although growth performance of the composite mutants (*dph2*Δ *eft2*Δ, *dph4*Δ *eft2*Δ) clearly is compromised on solid media under thermal/chemical stress (Fig 2; S1 Fig), the single mutants (*dph2*Δ, *dph4*Δ, *eft2*Δ) appear to tolerate these conditions and if any, show milder phenotypes. Similarly, in liquid media and under standard cultivation temperature (30°C), single mutants (*dph2*Δ, *eft2*Δ) perform almost like the wild-type, whereas growth of the composite mutant (*dph2*Δ *eft2*Δ) is significantly affected (S2 Fig). This phenotype, which results from negative interaction between the *dph2*Δ and *eft2*Δ null-alleles, is particularly obvious in delayed onset of the exponential growth phase and associates with slower growth rates and later entry into stationary phase (S2 Fig). Based on OD_600_ values, cell numbers of the mutant lacking diphthamide and proper pools of EF2 (*dph2*Δ *eft2*Δ) eventually become reduced in comparison to wild-type and single mutants (*dph2*Δ, *eft2*Δ) (S2 Fig).

**Fig 4:**
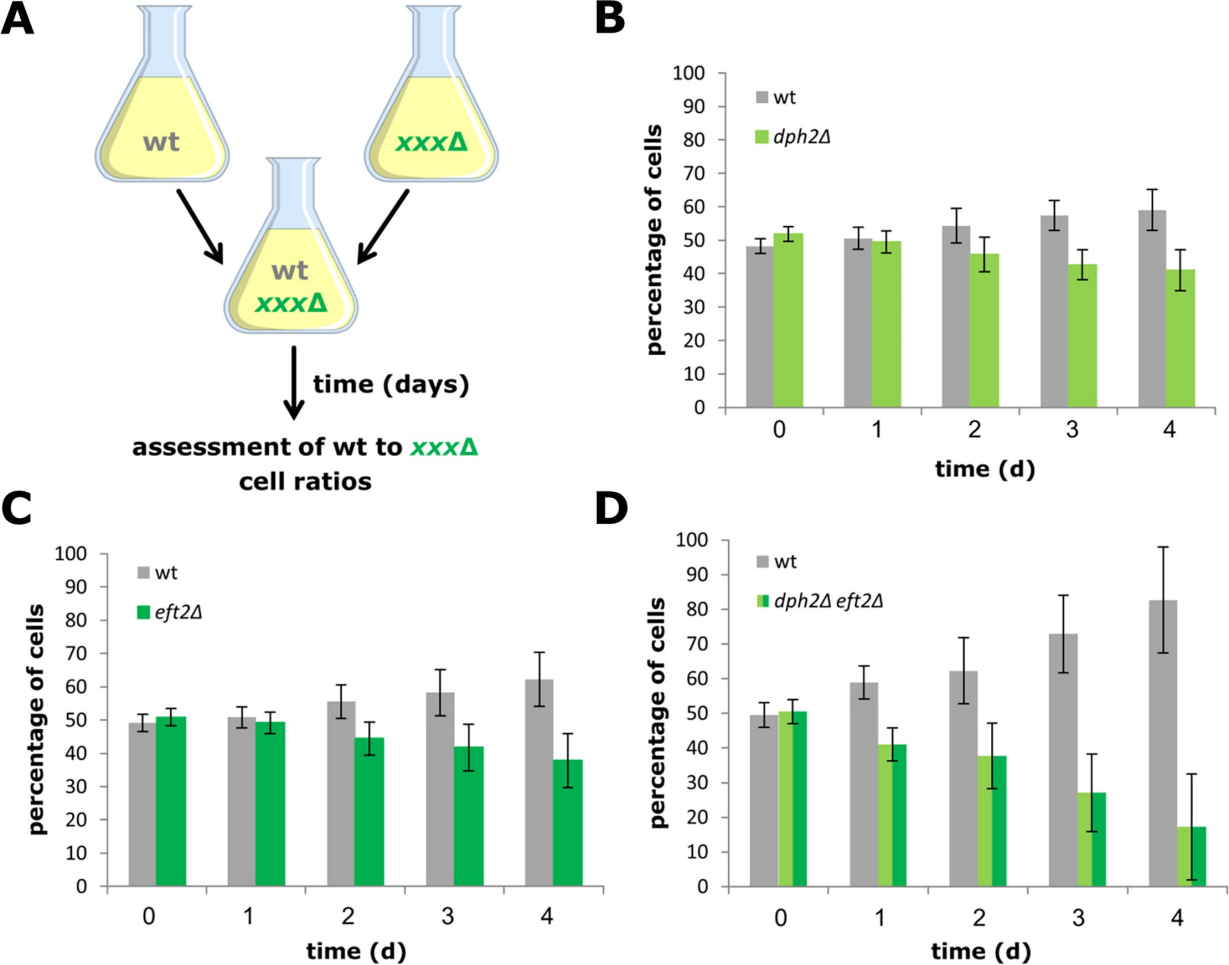
Competitive fitness studies in response to diphthamide defects, EF2 undersupply or both. (A) Experimental setup. Equal amounts of wild-type (wt) and mutant (xxxΔ) cells were mixed and grown for 24 h (1 d) and successively passaged three more times for a total period of 96 h (4 d). After each passage, the relative amount (% of cells) of the two cell-types was determined. Growth experiments involving competition between wt and *dph2*Δ (B), *eft2*Δ (C) or *dph2*Δ *eft2*Δ (D) cells are shown.

To further address the physiological importance of diphthamide modified EF2, we next examined competitive growth between mixed populations of wild-type and mutant (*dph2*Δ, *eft2*Δ, *dph2*Δ *eft2*Δ) cells (Fig 4A). The approach is based on previous observations that mixed cultures are well suited to detect subtle changes in the growth behaviour and competitive fitness between strains carrying wild-type alleles of non-essential genes and their respective deletions [50]. We found mutant cells that lack the ability to form diphthamide (*dph2*Δ) or that express reduced amounts of EF2 (*eft2*Δ) become outcompeted by wild-type cells after two days resulting in very similar cell reductions (*dph2*Δ: 41.0% ± 6.1%; Fig 4B and *eft2*Δ: 37.8% ± 8.1%; Fig 4C) after four days. In contrast, cells of the composite mutant (*dph2*Δ *eft2*Δ) display an additive fitness defect that is already obvious after one day and results in more drastic cell number reductions (17.3% ± 15.3%) after four days (Fig 4D). In sum, the synthetic fitness defects observed in our competition experiments go hand-in-hand with other growth-related phenotypic studies. They reinforce that subtle or minor traits in the single mutants (*dph2*Δ or *eft2*Δ) add up when genetically combined in the double mutant (*dph2*Δ *eft2*Δ) and compromise the cell performance as a result of lower-than-normal pools of EF2 that are not modifiable with diphthamide. The data therefore emphasize that diphthamide modified EF2 is important for competitive fitness and healthy yeast cells.

### Combinined diphthamide and EF2 defects alter growth rates and cell doubling times

**Fig 5:**
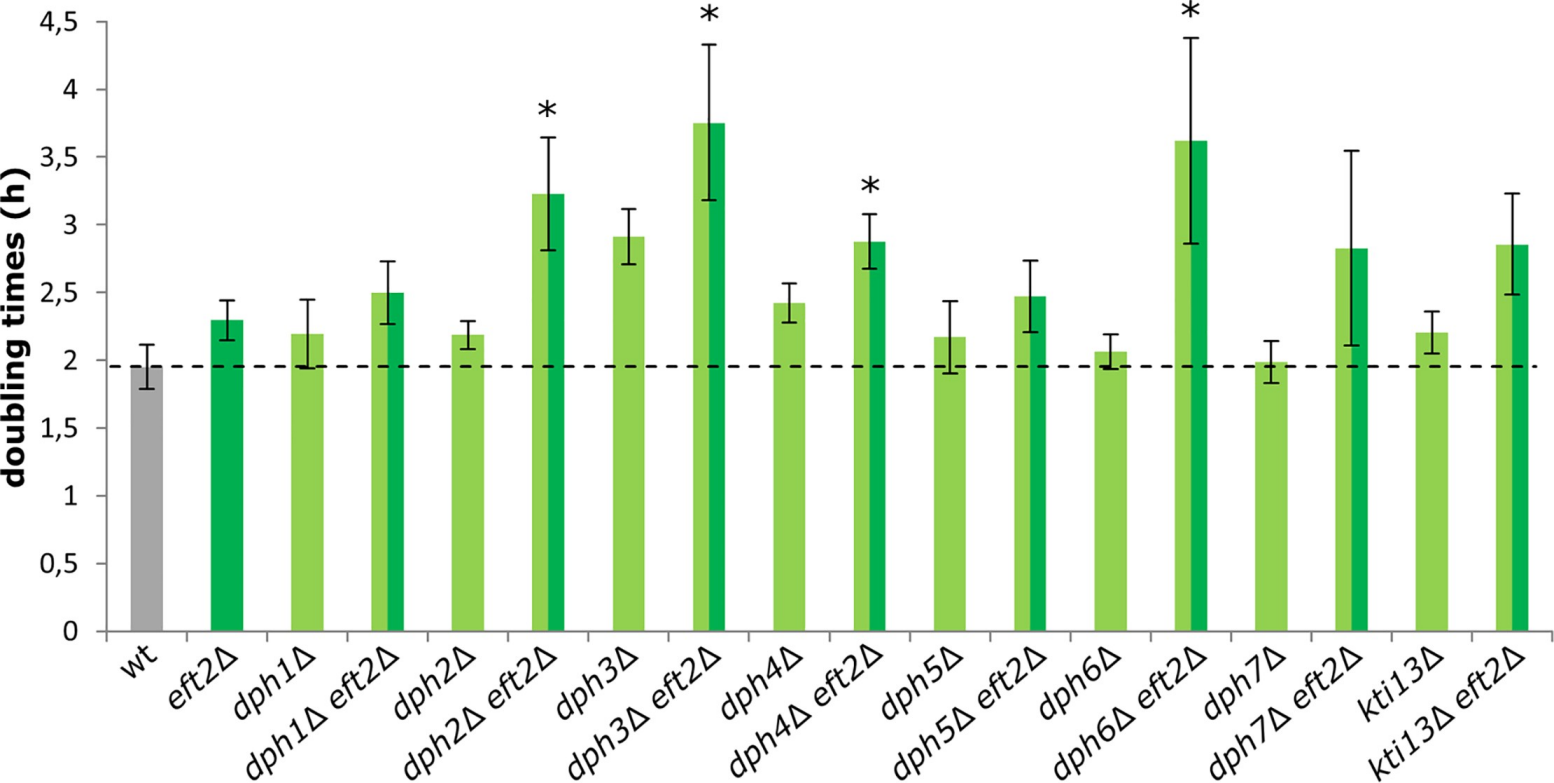
Analysis of cell doubling times in diphthamide synthesis (*DPH1-DPH7*) and *EFT2* gene deletion strains. Doubling times of the indicated strain backgrounds were determined from biological quadruplicates. Statistical significance was determined by two-tailed t-test. * (P ≤ 0.05).

To examine whether apart from *DPH2*, all other diphthamide biosynthesis genes (Fig 1) interact with *EFT2*, we combined each *dph*Δ mutant with *eft2*Δ null-alleles and performed (n = 4) single batch growth experiments for 23 h. The resulting exponential phases from each growth curve (for a representative read-out, see S2 Fig) were used to calculate the doubling times for each strain (Fig 5). Consistent with the predictions from TheCellMap.org [43] that members of the *DPH* gene network correlate with *EFT2*, we found that the doubling times in respective composite mutants (*dph2*Δ *eft2*Δ, *dph3*Δ *eft2*Δ,*dph4*Δ *eft2*Δ and *dph6*Δ *eft2*Δ) significantly increased in relation to the single mutants (*dph*Δ and *eft2*Δ) alone (Fig 5). Furthermore, similar to the strength of negative genetic interaction coefficients extracted from TheCellMap.org (Fig 2B), doubling times of *dph2*Δ and *dph6*Δ mutants scored significantly higher in tandem with *eft2*Δ alleles, while EF2 undersupply reduced the doubling times of *DPH1*, *DPH4*, *DPH5* and *DPH7* deletion strains to a lesser but significant degree (Fig 5).

*DPH3*, originally identified iso-allelic with *KTI11* [51,52], is absent from the gene deletion collection used for SGA assembly of TheCellMap.org [43]. This explains why prior to our study here, no *DPH3/KTI11* and *EFT2* correlation was reported. Strikingly, reduction in the doubling times of the composite mutant (*dph3/kti11*Δ *eft2*Δ) was found to be even stronger than in *dph2*Δ *eft2*Δ and *dph6*Δ *eft2*Δ (Fig 5) mutants most strongly correlated by the SGA criteria above (Fig 2). Presumably, this is due to roles for *DPH3/KTI11* in both diphthamide synthesis and the Elongator pathway for tRNA anticodon modification [6,25,53–55]. In line with such functional difference from other *dph*Δ mutants, a significant doubling time increase is already observed with the single (*dph3/kti11*Δ) mutant alone (Fig 5), which is pleiotropic *per se* due to defects in the diphthamide and tRNA modification pathways [6,53]. Additional EF2 undersupply in the genetic background of the composite (*dph3/kti11*Δ *eft2*Δ) mutant may therefore further compromise its translational capacity. Recently, Kti13, another protein involved in the Elongator pathway for tRNA modification [51,55–57] was proposed to play a role in diphthamide synthesis through complex formation with Dph3/Kti11 [6,53,54].

In support of this, we score negative genetic interaction based on significantly reduced cell doubling times upon combining *KTI13* and *EFT2* deletions, and the effect is similar to most of the *bona fide* (*dph*Δ *eft2*Δ) composite mutants (Fig 5). In sum, our data are in further support that the *DPH1-DPH7* and *EFT2* gene networks functionally correlate and that combined deletions in the composite mutants (*dph*Δ *eft2*Δ) cause synthetic effects. This indicates that under conditions of EF2 undersupply (*eft2*Δ), either absence (*dph1*Δ*-dph4*Δ) or incomplete synthesis (*dph5*Δ*-dph7*Δ) of diphthamide on EF2 compromise the essential function of the translation factor and trigger defects in cell growth, viability and fitness.

### Loss of diphthamide modifiable EF2 pools confers *DPH5* overexpression toxicity

**Fig 6:**
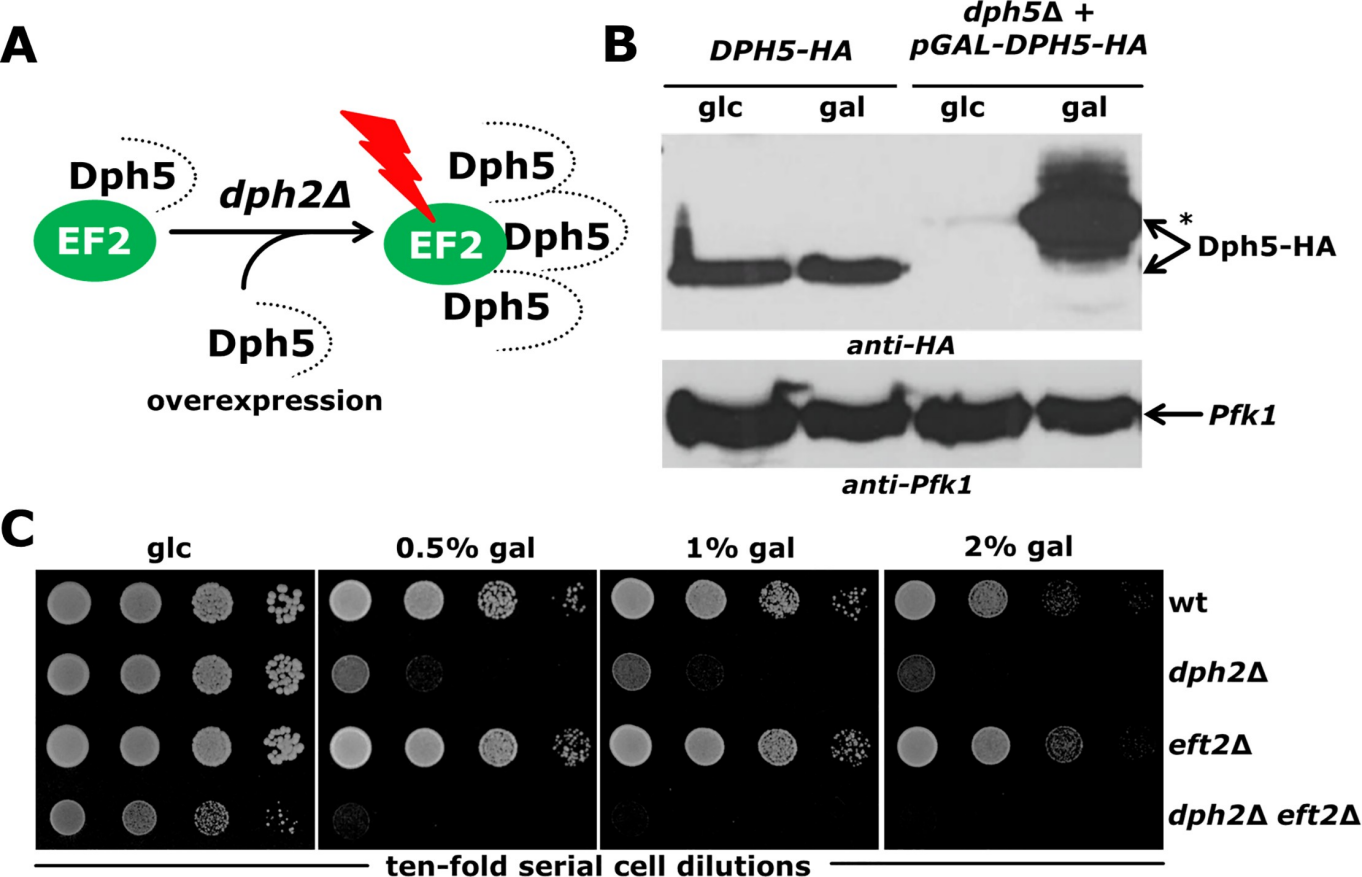
*DPH5* overexpression toxicity. (A) Model depicting that higher-than-normal Dph5 levels interact with EF2 when diphthamide synthesis is absent (*dph2*Δ) and can become EF2 inhibitory (red bolt) and cytotoxic upon *DPH5* overexpression [13,19,24,58]. (B) Expression analysis between Dph5-HA produced from the wild-type genomic locus (*DPH5-HA*) and a *GAL*-promoter plasmid (p*GAL-DPH5-HA*) (in a *dph5*Δ strain). Protein extracts obtained after growth on glucose (glc) or galactose (gal) medium were analyzed by anti-HA (top panel) or anti-Pfk1 (bottom panel) Western blots to detect Dph5-HA or phosphofructokinase expression levels. Dph5-HA produced from p*GAL-DPH5-HA* carries additional tags [59] that confer a mobility-shift (*). (C) *DPH5* overexpression is toxic for growth of *dph2*Δ and *dph2*Δ *eft2*Δ mutants. The indicated strains were cultivated for 3 days on glc (2% [w/v]) and gal (0.5, 1, 2% [w/v]) medium.

Prior to ACP formation by Dph1-Dph4, the methylase Dph5 uniquely binds to unmodified EF2 and later dissociates from methyl-diphthine (Fig 1) in a Dph7 dependent manner [19,58]. In cells with incomplete (*dph7*Δ) or absent (*dph1*Δ*-dph4*Δ) diphthamide synthesis, excess Dph5 levels were found to accumulate on EF2 (Fig 6A) and in response to upregulated *DPH5* gene activation from the strong *GAL* promoter to inhibit cell growth [13,19,24,58]. To correlate Dph5 levels under wild-type (native *DPH5* promoter) and ectopic (*GAL* promoter) expression regimes, we compared Dph5 production from genomic [13] and plasmid-borne [59] *DPH5-HA* alleles in Western blots using the anti-HA antibody. As shown in Fig 6B, ectopic Dph5-HA expression in a *dph5*Δ reporter strain is virtually shut-down by glucose and drastically induced by galactose in comparison to constitutive Dph5-HA levels produced from the wild-type locus and irrespective of carbon source supply. Thus, *DPH5* overexpression and production of higher-than-normal Dph5 levels most likely account for the cytotoxicity typically observed with *dph1*Δ*-dph4*Δ mutants [13,19,24].

In an attempt to further diagnose synthetic interaction between EF2 and diphthamide defects, we therefore analysed toxicity of overexpression of *DPH5* in *dph2*Δ, *eft2*Δ and *dph2*Δ *eft2*Δ mutants. While the performance of the mutant with reduced EF2 copy number (*eft2*Δ) hardly differs from the wild-type with a full genetic EF2 complement, *DPH5* overexpression inhibits growth of the diphthamide mutant (*dph2*Δ) alone and even more in combination with EF2 undersupply (*dph2*Δ *eft2*Δ) (Fig 6C). Moreover, *DPH5* overexpression toxicity is modulated in a galactose dose dependent fashion and strongest in the composite mutant (*dph2*Δ *eft2*Δ) lacking modifiable pools of EF2 (Fig 6C). We conclude that rather than EF2 undersupply (*eft2*Δ), it is loss of diphthamide (*dph2*Δ) which conditions growth inhibition by excess levels of Dph5 and enhances cytotoxicity in tandem with EF2 undersupply (*dph2*Δ *eft2*Δ) (Fig 6C). Again, our data support synthetic negative genetic interactions showing that both EF2 and diphthamide correlate and that the diphthamide modification is critical for full functioning of translation factor EF2.

### Elevated -1 frame-shift errors results from loss of diphthamide modified EF2 pools

**Fig 7:**
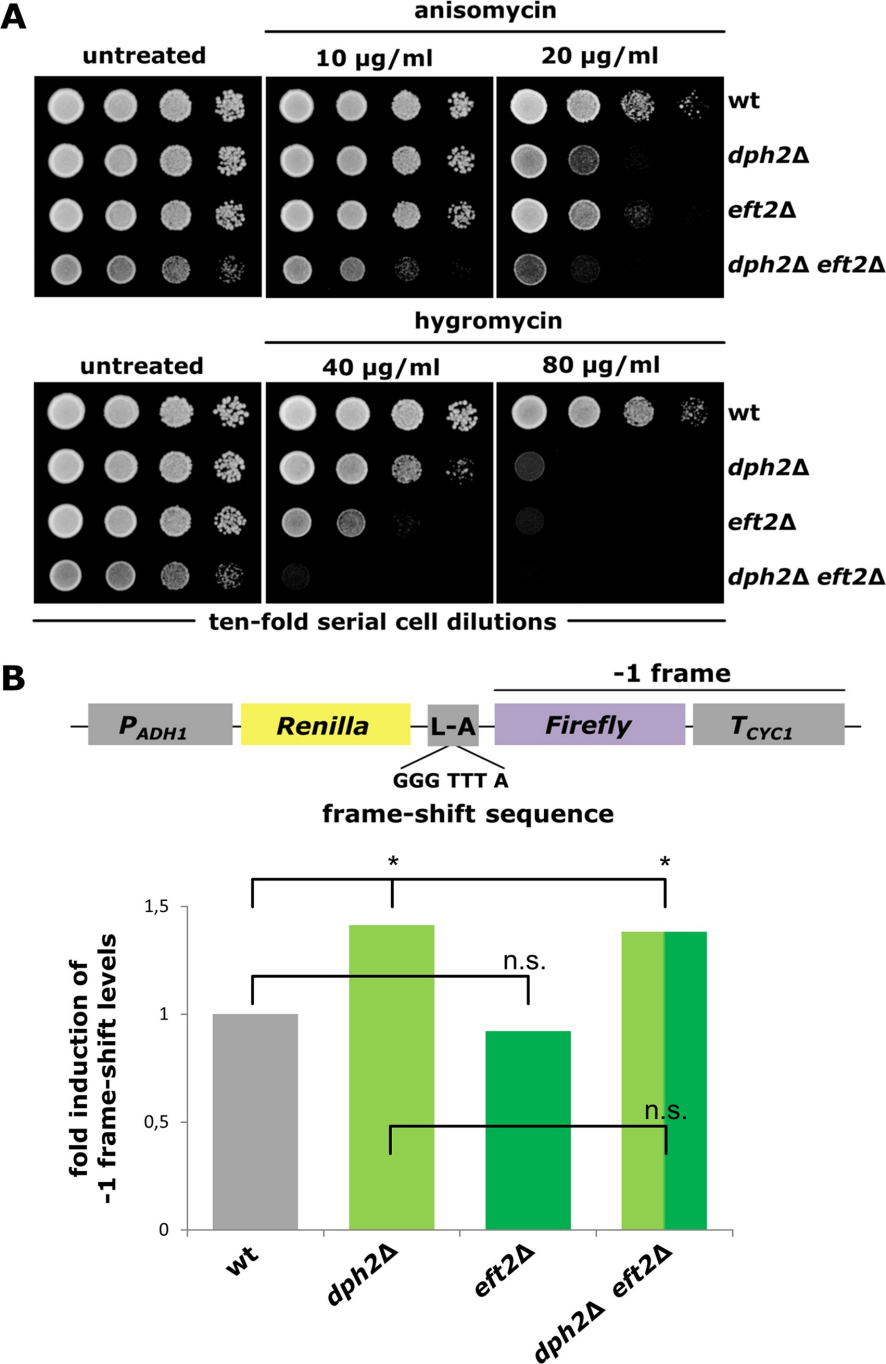
Growth response to translation indicator drugs and vulnerability to programmed -1 frame-shifting of mutants lacking diphthamide and/or proper EF2 supply (*dph2*Δ, *eft2*Δ and *dph2*Δ *eft2*Δ). (A) Serial cell dilutions of the indicated yeast stains were cultivated on media without any drugs (untreated) or supplemented with various doses of anisomycin (10, 20 μg/ml) or hygromycin (40, 80 μg/ml) at 30°C for 2–3 days. (B) Programmed -1 frame-shift assays based on a dual luciferase reporter system (scheme) carrying a viral -1 frame-shift site: 5’-GGGTTTA-3’ [62]. Luminescence based read-outs were statistically analyzed using two-tailed t-test. * (P ≤ 0.05); n.s. (P > 0.05).

Our data strongly suggest a physiological role for diphthamide in cell viability, which is likely related to the translation elongation activity of EF2. We therefore studied whether the synthetic phenotypes in mutants with reduced EF2 gene copy number and diphthamide defects (*dph*Δ *eft2*Δ) may result from inaccurate or error-prone translation. Growth assays performed in the presence of hygromycin and anisomycin revealed inhibition of two composite mutants (*dph2*Δ *eft2*Δ and *dph4*Δ *eft2*Δ) in a dose dependent manner by these translation indicator drugs (Fig 7A; S3 Fig).

This implies that lack of diphthamide in tandem with EF2 undersupply compromises a cell’s translational capacity and thus enhances its vulnerability to translation elongation targeting antibiotics. In line with this notion, which suggests that diphthamide assists EF2 during mRNA translation, previous studies show that substitutions in EF2 diphthamide acceptor site His-699 or other conserved residues nearby confer problems in reading frame maintenance [32,47,60,61]. Hence, we investigated the propensity of our mutant set (*dph2*Δ, *eft2*Δ and *dph2*Δ *eft2*Δ) to induce -1 ribosomal frame-shift errors using a plasmid based dual luciferase reporter system previously established by Harger & Dinman (2003) [62]. Monitoring programmed -1 frame-shift errors on the basis of the L-A viral reporter sequence [62], we observed that a deletion of *DPH2* alone (*dph2*Δ) leads to elevated -1 frame-shift errors, while reduced amounts of EF2 as such (*eft2*Δ) cause no effect on ribosomal accuracy and reading frame maintenance (Fig 7B). Intriguingly, the composite (*dph2*Δ *eft2*Δ) mutant is also found to be error-prone with detectable -1 frame-shift frequencies that are similar, if not identical, to the single mutant (*dph2*Δ) alone (Fig 7B). We conclude that EF2 undersupply alone does not induce -1 frame-shift errors, while loss of diphthamide modification compromises the translational accuracy of EF2. Taken together, our data strongly suggest that the synthetic growth defects typical of the composite mutants (*dph*Δ *eft2*Δ) highly likely result from error-prone translation (*dph*Δ) in combination with reduced translational capacity of EF2 (*eft2*Δ). This corresponds well with previous observations on translational errors associated with lack of diphthamide [32,47,60,61] and reinforces our view that the modification of EF2 is critical for accurate functioning of the translation factor.

## Discussion

Previously, genome-wide approaches based on chemical genomics [FitDB] and SGA [DRYGIN] databases from *S*. *cerevisiae* enabled discovery of *DPH6* and *DPH7* [13,16–19], two hitherto unknown genes of the biosynthetic pathway for modification of EF2 by diphthamide. The approach allowed elucidation of new biochemical activities in the terminal steps of diphthamide synthesis (Fig 1) and provided robust phenotypic signatures indicative for a tightly clustered *DPH1-DPH7* network within the genetic interaction landscape of yeast [13,16–19]. The present study further exploits the SGA database update (TheCellMap.org) showing strong correlation between the diphthamide (*DPH1-DPH7*) and EF2 (*EFT1-EFT2*) gene networks. Thus, our SGA-prompted study served as a starting point to further delineate the physiological role of diphthamide, which unlike its well-known pathological role as a target for ADP ribosylase toxins [13,14], is less clear.

Our analysis is diagnostic for synthetic genetic interactions in most of the combined EF2 and diphthamide gene deletion strains. With the exception of *DPH3*/*KTI11* (for which there are no SGA database entries), this notion is based on stringent PCC values between pairwise *EFT2* and *DPH* gene deletions (Fig 2A) and strong negative interaction between an *EFT2* query gene deletion and *DPH1-DPH7* null-alleles from the array collection (Fig 2B). Intriguingly, we find that the EF2 gene *EFT1*, which is essential for a viable *eft2*Δ mutant [47], contributes to ~ 35% of total EF2 and thus plays a minor role compared to the *EFT2* paralog. Lower EF2 levels produced from *EFT1* possibly explain why (among the two EF2 gene copies) *EFT2* is stronger correlated with each member of the *DPH1-DPH7* gene network (Fig 2A).

Irrespective of differential EF2 levels, the correlation data from SGA imply that the EF2 and diphthamide gene networks are linked to each other in function. This notion is based on synthetic interactions and entirely goes hand-in-hand with previous evidence from co-immune precipitation, pull-down experiments, TAP purification and catalytic studies, which collectively demonstrate biophysical interactions *in vivo* and *in vitro* between EF2 and the diphthamide machinery [6,16–25,29,31,52]. In addition, based upon phenotypic, genetic and biochemical criteria, we observe mutual interactions in our experimental assays. Thus, by comparing the performance of mutants with diphthamide defects (*dph*Δ), EF2 undersupply (*eft2*Δ) or a combination of both (*dph*Δ *eft2*Δ) to cells with proper pools of diphthamide modified EF2, we confirm the above SGA based interactions. While the single mutants (*dph*Δ, *eft2*Δ) alone hardly display any growth-related phenotypes, the composite (*dph*Δ *eft2*Δ) mutants undergo additive growth defects that specifically arise from combining EF2 undersupply with loss of diphthamide modification. Inappropriate pools of EF2 which are not diphthamide modified in the double mutant (*dph2*Δ *eft2*Δ) could be validated by MS measurements and Western blot analysis used to examine both the levels and diphthamide modification states of EF2. Here, *anti-EF2(no diphthamide)*, an antibody previously established [33] to recognize human EF2 that is not diphthamide modified was also found to detect exclusively unmodified EF2 in diphthamide mutants (*dph2*Δ, *dph2*Δ *eft2*Δ) from yeast. Cross-species specificity likely results from conservation of eukaryal EF2, and the peptide sequence derived from human EF2 for antibody generation [33] is indeed identical to the yeast counterpart (see Materials & Methods section). Hence, we consider the *anti-EF2(no diphthamide)* Western blot provides a facile means to readily detect diphthamide modification states of EF2 and potentially distinguish diphthamide intermediates from individual pathway (Fig 1) mutants.

In addition, our *anti-EF2(pan)* Western blots and MS data indicate slight but significantly (~1.3 fold) increased EF2 levels in diphthamide (*dph2*Δ) minus cells. Considering the negative genetic interactions that result from loss of diphthamide and reduced EF2 downregulation, it seems reasonable for us to speculate that EF2 upregulation may be a cellular response to help rescue from phenotypes that become particularly apparent under conditions of EF2 undersupply in diphthamide mutants (*dph*Δ *eft2*Δ). Indeed, we present in here a robust body of evidence showing that the latter scenario in the composite mutants (*dph*Δ *eft2*Δ) dramatically affects (i) cell performance under standard condition as well as thermal and/or chemical stress, (ii) cellular growth rates, (iii) cell doubling times, (iv) competitive fitness, (v) *DPH5* overexpression toxicity and (vi) cell viability in the presence of TOR inhibitor (rapamycin, caffeine) and translation indicator drugs (hygromycin, anisomycin).

This is a plethora of synthetic negative phenotypes indicating that the diphthamide modification on EF2 promotes cell growth and competitive fitness, most likely through effects on mRNA translation. In line with this notion, our data show that it is loss of diphthamide modified EF2 (*dph2*Δ) rather than reduced levels of the translation factor (*eft2*Δ), which triggers growth inhibition by higher-than-normal Dph5 levels produced from *DPH5* overexpression. With excess levels of Dph5 being previously shown to bind and inhibit EF2, particularly when unmodified [13,19,24,58], cytotoxicity is expected to further increase when EF2 levels become limiting. This is exactly what we see in the composite mutant (*dph2*Δ *eft2*Δ), and we therefore speculate that Dph5 –in addition to its catalytic activity as a promiscuous tetra-methylase [63] (Fig 1)–can negatively interfere with EF2 function and cell growth when the stepwise generation of diphthamide on EF2 is absent (*dph1*Δ-*dph4*Δ) or incomplete (*dph7*Δ) [13,19,24,58]. Perhaps, such a regulatory rather than catalytic role for Dph5 involves binding to EF2 in order to exclude the elongation factor from functioning in mRNA translation unless licensed for full modification with diphthamide [11,13].

Moreover, the diphthamide modification becomes particularly important and even essential for cell viability under conditions that limit EF2 function and as a result, compromise a cell’s translational capacity. Consistently, we find growth inhibition by anisomycin and hygromycin, two aminoglycosidic antibiotics that interfere with eukaryal mRNA translation by inhibiting peptidyl transferase activity, disrupting EF2 dependent translocation and promoting mistranslation [64], is most aggravated in the composite mutants (*dph2*Δ *eft2*Δ, *dph4*Δ *eft2*Δ) lacking diphthamide and proper levels of EF2. Together with -1 frame-shift data showing that diphthamide modified EF2 significantly contributes to reading frame maintenance and translational accuracy, we propose that the synthetic growth phenotypes typical of the composite mutants (*dph*Δ *eft2*Δ) are most likely the result of impaired translational capacity and error-prone translation.

In support of this model, which implies direct or indirect involvement of diphthamide in ribosomal decoding, the location of the diphthamide modification at the tip of anticodon mimicry domain IV in EF2 predicts a potential role in mRNA translation [60]. Consistent with this are structure-function studies showing that domain IV may be sufficiently close to interact with tRNA in the ribosomal P-site [60]. In addition, substitutions of highly conserved tip residues in domain IV, including diphthamide acceptor site His-699 and critical mutations nearby (H694N, I698A, P580H) were shown to trigger -1 frame-shifts in yeast [32,40,61].

Although these studies cannot clarify whether reading frame maintenance is a general function of EF2 or specific to its diphthamide modified tip, there is recent structural evidence showing that the modification links up with mRNA and rRNA in the ribosomal decoding centre [41]. Solving the structure at near atomic resolution, the report [41] concludes that through ensuring reading frame maintenance, diphthamide plays a role in ribosomal translocation during the elongation phase of mRNA translation. Similarly, cryo-EM based structural studies have emerged suggesting that diphthamide may stabilize the interaction of the codon-anticodon duplex and thereby prevent from reverse translocation activity of EF2 [42]. Interestingly, reducing the interactions of the anticodon mimicry domain IV on EF2 with the codon-anticodon helix by loss of diphthamide [42,65,66] could explain the increase in -1 frame-shift errors that research groups including our own have observed in mutants that lack diphthamide modified EF2 or fail to complete diphthamide synthesis [19,32,40,61,67]. Interestingly, a neurological disorder in humans (SCA26) was found to result from a mutation in EF2 that affects a residue close to the diphthamide modified histidine in the 3D structure of EF2 and also induces -1 frameshifting [67]. In sum, our *in vivo* experiments together with recent structural and biochemical studies strongly suggest that the role of the diphthamide modification in EF2 is to optimize the efficiency and accuracy of EF2 in ribosomal translation [41,42,68].

With dual functions of *DPH3*/*KTI11* in diphthamide modification of EF2 [6,15,25] and Elongator dependent tRNA modification [53–55], it will be important to test whether both pathways cooperate with one another as has been suggested in previous studies on telomere biology and stress responses [69,70]. The latter includes shared sensitivity to TOR inhibitor drugs (rapamycin, caffeine) of Elongator mutants [71] and our composite mutants (*dph2*Δ *eft2*Δ, *dph4*Δ *eft2*Δ) (Fig 3C and S1 Fig). As for the Elongator pathway, there is evidence for tRNA modification cross-talk that promotes proper codon translation rates and reading frame maintenance [72–77] similar to our data in here indicating that translational fidelity and efficiency are supported by diphthamide modified EF2.

## Materials and methods

### Strains, media, growth conditions and assays

Yeast strains used and generated in this study are listed in S1 Table. Cultures were grown in complete (YPD) or minimal (SD) media [78] at 30°C unless otherwise stated.

For phenotypic analysis, cell solutions (OD_600_ of 1.5) were ten-fold serially diluted and spotted onto YPD plates [78] containing SDS [0.2% w/v], rapamycin [15–30 nM], caffeine [7.5–10 mM], anisomycin [10–20 μg/ml], hygromycin [40–80 μg/ml] or no drug, respectively, and incubated at 30°C for 2–3 days. Thermal stress tolerance was tested by incubating plates under conditions of elevated temperature cultivation (37°C, 39°C and 40°C). *DPH5* overexpression cytotoxicity assays utilized the galactose inducible plasmid p*GAL-DPH5-HA* from the mORF overexpression library [59]. p*GAL-DPH5-HA* carrying yeast strains were selected following transformation with the lithium acetate protocol [79] on SD medium lacking uracil, and growth assays were conducted on the same medium containing glucose (2% [w/v]) or galactose (0.5, 1, 2% [w/v]) essentially as described [19].

### Generation of yeast gene deletion strains and growth competition assays

Yeast strains carrying single or double *DPH1-DPH7* and *EFT2* gene deletions were constructed using plasmid (pUG, YDp) based PCR knock-out cartridges [80, 81] and primer pairs specific for each genomic manipulation listed in S2 Table. All gene deletions were confirmed via diagnostic PCR on genomic DNA preparations using target gene specific primer pairs (S2 Table). For the growth competition experiments (Fig 4), yeast cells were pre-grown in single batch liquid YPD medium for one day at 30°C before mixtures of equal amounts (OD_600_ = 0.1) of wild-type control and mutants (*dph2*Δ, *eft2*Δ and *dph2*Δ *eft2*Δ) were used to inoculate the main cultures. These were grown for 24 h before a portion (1:1000) was transferred into fresh medium. This process was repeated four times and every day, a portion (1:100,000) of the cells was plated on YPD medium and YPD containing 400 μg/ml of the antibiotic G418. Plates were incubated at 30°C for 2 days to select for and differentiate between wild-type and mutant cells. The resulting relative ratios of wild-type and mutant cells were progressively monitored over a total of four days.

### Dual-luciferase -1 frame-shift assay

Using the lithium acetate protocol [79], yeast cells were transformed with dual-luciferase control (pJD375) and -1 frame-shift reporter (pJD376) plasmids [62] and grown overnight in SD medium lacking uracil. After cell lysis, analysis of luciferase activities involved the dual-luciferase reporter assay system (Promega; cat. no. E1910) and measurements with a luminometer (GloMax Explorer Multimode Microplate Reader - Promega; cat. no. GM3500). At least 13 individual biological replicates were used for frame-shift analysis of each strain. Frame-shift error percentages were calculated in relation to control strains carrying pJD375. Frame-shift rates were normalized to wild-type levels and statistical significance was verified by using the two-tail t-test.

### Epitope-tagging and protein detection by Western blots

C-terminal HA-tagging of *DPH5* was performed according to previously published *in vivo* PCR based manipulation protocols [6,19]. The tagged gene (*DPH5-HA*) and its product (Dph5-HA) were confirmed respectively, by diagnostic PCR and Western blots using the anti-HA antibody A-14 (Santa Cruz Biotechnology) according to the manufacturer’s recommendations. Detection of global EF2 irrespective of its diphthamide modification state, used Western blots with antibody *anti-EF2(pan)* (3C2) while immune blots with the antibody *anti-EF2(no diphthamide)* (10G8) [33] solely detected pools of EF2 without the diphthamide modification. Antibodies 3C2 and 10G8 were previously generated to detect human EF2 [33]. Since the sequence recognized by 10G8 is identical between human (_708_TLHADAIHRGGGQIIPT_724_) and fungal (_692_TLHADAIHRGGGQIIPT_708_) EF2 [33], it can also be applied to differentiate diphthamide modification states of EF2 from yeast. Total cell extracts were generated from human MCF7 *DPH2* knock-out cell-lines (*dph2*ΔΔ: Western blot control) by lysing 4.5x 10^5^ cells on ice in 150μl RIPA buffer (Sigma, cat. no. R0278) containing protease inhibitor ‘complete’ (Roche; cat. no. 11697498001; 1 tablet/10ml buffer). Total yeast cell extracts were generated from strains grown to OD_600_ of 1, isolated via glass bead lysis [82] and protein concentrations were determined by Bradford assay [83]. 7.5μl of MCF7 or yeast extracts (including loading buffer) were subsequently subjected to reducing SDS-PAGE and blotted onto PVDF membranes (Merck/Millipore; cat. no. IPVH07850). The membranes were probed overnight at 4°C with antibodies 3C2 or 10G8 [33] and detected/visualized with anti-rabbit secondary antibody HRP-conjugate (Dako; cat. no. P0217 –working conc. 1:2000) and Lumi-Light Western blotting substrate (Roche; cat. no. 12015200001) as previously described [33]. For MCF7 cells, protein loading was controlled in Western blots with an antibody against β-actin (Sigma; cat. no. A2228 –working conc. 1:1500). For yeast, this utilized anti-Pfk1 or anti-Cdc19 antibodies kindly donated by Jürgen Heinisch (University of Osnabrück, Germany) and Jeremy Thorner (University of California, USA), respectively.

### Protein identification and quantification by mass spectrometry

Gel pieces containing 40 μg of whole protein extracts from the respective mutants were first amended with glyceraldehyde-3-phosphate dehydrogenase (GAP-DH) of *Staphylococcus aureus* as an internal standard. Then the gel-fixed proteins were reduced with dithiothreitol, sulfhydryl-groups were derivatized with iodoacetamide and proteins were digested with trypsin as described [84]. The resulting peptide mixtures were desalted with ZipTip-μC18 material (Merck Millipore) and analyzed with an Ultimate 3000 nanoRSLC instrument (Thermo Scientific, Germering, Germany) coupled to an Orbitrap Fusion mass spectrometer (Thermo Scientific, San Jose, CA, USA) via a TriVersa NanoMate (Advion, Ltd., Harlow, UK) as described previously [85]. Proteins were identified by matching precursor and fragment mass spectra against the NCBI protein database of *Saccharomyces cerevisiae* strain S288C using SequestHT as a built-in search engine of ProteomeDiscoverer version 2.2 (Thermo). For identification, a mass tolerance of 3 ppm for precursor ions and 0.1 Da for fragment ions was set. Elongation factor 2 (EF2) was detected in all analyzed samples with an average coverage of almost 80%. EF2 quantification was done on the basis of intensity values that were normalized against the internal standard.

## Supporting information

S1 File SGA data for *DPH1-DPH7* and *EFT1-EFT2* query genes based on TheCellMap.org database mining. (XLS)

S1 Fig Synthetic sick growth phenotypes result from combining EF2 downregulation with loss of diphthamide. Ten-fold serial cell dilutions of wild-type (wt), single (*dph4*Δ and *eft2*Δ) and composite (*dph4*Δ *eft2*Δ) mutants were cultivated at different temperatures (30°C, 37°C, 39°C or 40°C) or incubated in the absence (untreated) or presence of various chemical stressors (rapamycin [15–30 nM] or caffeine [7.5–10 mM]).
(TIF)

S2 Fig Negative interactions between EF2 and diphthamide gene deletions in composite mutant (*dph2*Δ*eft2*Δ) reduce growth and doubling times. Shown are growth curves of wild-type (wt), single (*dph2*Δ and *eft2*Δ) and double mutant (*dph2*Δ *eft2*Δ) cells monitored over a period of 23 h in liquid rich (YPD) medium. Cells were cultivated in single batches of 50 ml medium and optical densities were measured at OD_600_ nm every hour. While *dph2*Δ or *eft2*Δ cells hardly differ in relation to wt cells, the double *dph2*Δ *eft2*Δ mutant is significantly reduced in growth.
(TIF)

S3 Fig Hygromycin phenotypes of mutants lacking diphthamide and/or proper EF2 supply. Serial cell dilutions of the indicated yeast stains were cultivated on media without (untreated) or supplemented with various hygromycin doses (40, 80 μg/ml) at 30°C for 2–3 days.
(TIF)

S1 Table Yeast strains used or generated for this study. (DOCX)

S2 Table Primers used in this study. (DOCX)
