# Supplementary figures and images for: Importance of diphthamide modified EF2 for translational accuracy and competitive cell growth in yeast
Source: PLoS One. 2018 Oct 18;13(10):e0205870. doi: 10.1371/journal.pone.0205870 (PMC6193676; doi:10.1371/journal.pone.0205870)

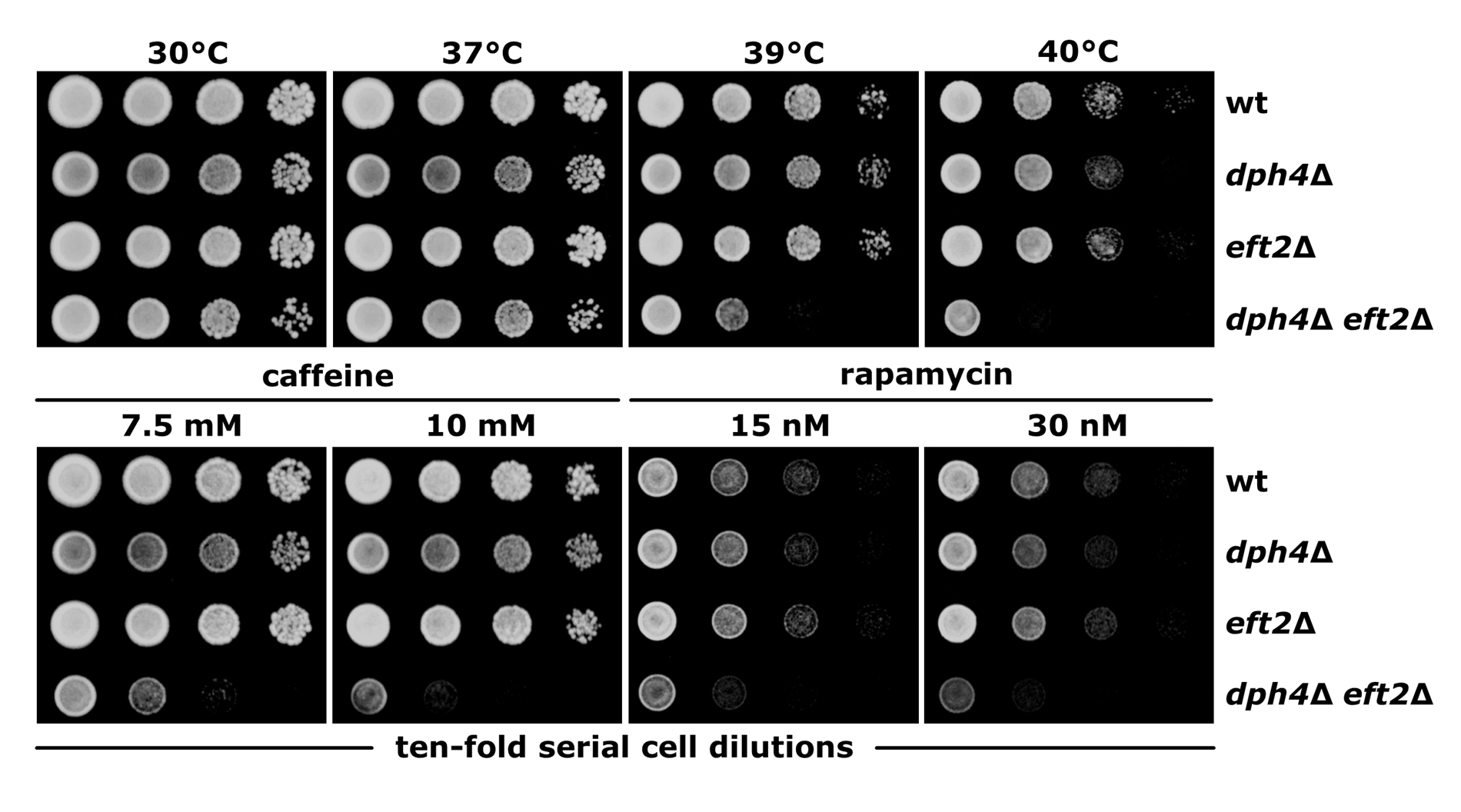

Supplement: S1 Fig — Ten-fold serial cell dilutions of wild-type (wt), single (dph4Δ and eft2Δ) and composite (dph4Δ eft2Δ) mutants were cultivated at different temperatures (30°C, 37°C, 39°C or 40°C) or incubated in the absence (untreated) or presence of various chemical stressors (rapamycin [15–30 nM] or caffeine [7.5–10 mM]). (TIF) [file pone.0205870.s002.tif]

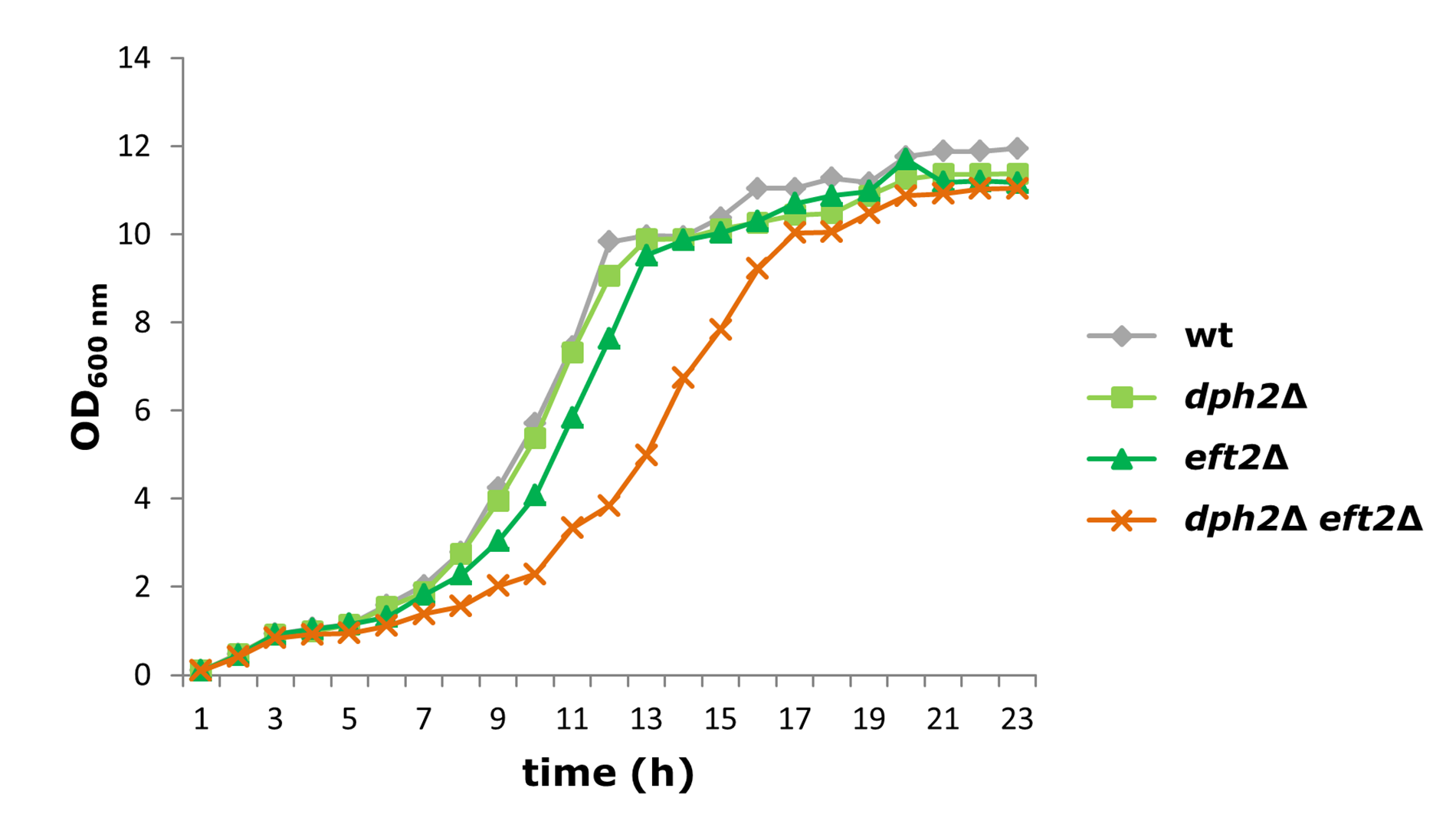

Supplement: S2 Fig — Shown are growth curves of wild-type (wt), single (dph2Δ and eft2Δ) and double mutant (dph2Δ eft2Δ) cells monitored over a period of 23 h in liquid rich (YPD) medium. Cells were cultivated in single batches of 50 ml medium and optical densities were measured at OD600 nm every hour. While dph2Δ or eft2Δ cells hardly differ in relation to wt cells, the double dph2Δ eft2Δ mutant is significantly reduced in growth. (TIF) [file pone.0205870.s003.tif]

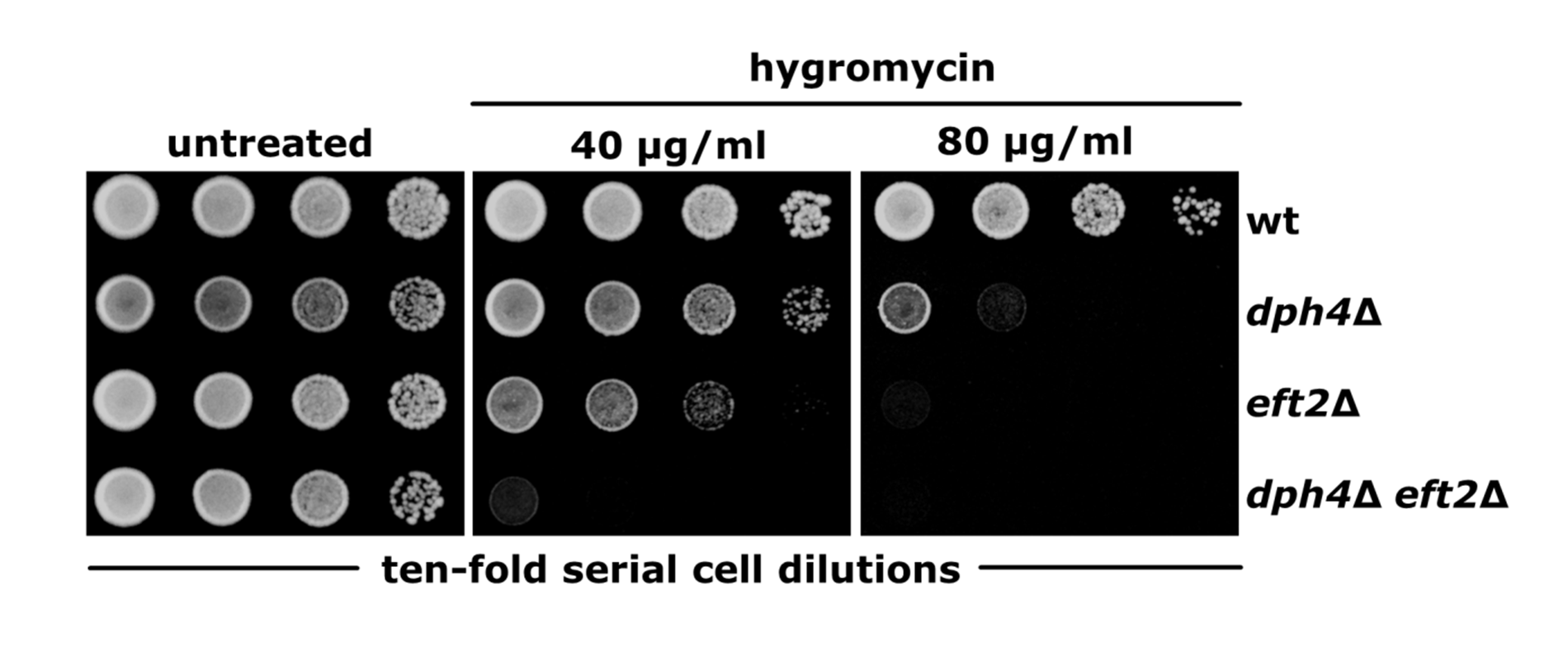

Supplement: S3 Fig — Serial cell dilutions of the indicated yeast stains were cultivated on media without (untreated) or supplemented with various hygromycin doses (40, 80 μg/ml) at 30°C for 2–3 days. (TIF) [file pone.0205870.s004.tif]
