# Supplementary material for: Importance of diphthamide modified EF2 for translational accuracy and competitive cell growth in yeast
Source: PLoS One. 2018 Oct 18;13(10):e0205870. doi: 10.1371/journal.pone.0205870 (PMC6193676; doi:10.1371/journal.pone.0205870)
Supplement: S1 Table — (DOCX) [file pone.0205870.s005.docx]

**S1 Table. Yeast strains used or generated for this study.**

| Strain | Genotype | Source |
| --- | --- | --- |
| BY4741 | *MATa his3∆1 leu2∆0 met15∆0 ura3∆0* | Euroscarf |
| HH2 | BY4741, *dph1∆:: kanMX4* | Euroscarf |
| HH3 | BY4741, *dph2∆::kanMX4* | Euroscarf |
| CBY12 | BY4741, *dph3∆:: loxP::SpHIS5::loxP* | [1] |
| HH5 | BY4741, *dph4∆::kanMX4* | Euroscarf |
| HH6 | BY4741, *dph5∆::kanMX4* | Euroscarf |
| HH7 | BY4741, *dph6∆::kanMX4* | Euroscarf |
| HH8 | BY4741, *dph7∆::kanMX4* | Euroscarf |
| HH10 | BY4741, *kti13∆::kanMX4* | Euroscarf |
| HH14 | BY4741, *eft2∆::kanMX4* | Euroscarf |
| HH15 | BY4741, *dph1∆:: loxP::SpHIS5::loxP* | this study |
| HH16 | BY4741, *dph2∆:: loxP::SpHIS5::loxP* | this study |
| HH17 | BY4741, *dph4∆:: loxP::SpHIS5::loxP* | this study |
| HH18 | BY4741, *dph5∆:: loxP::SpHIS5::loxP* | this study |
| HH19 | BY4741, *dph6∆:: loxP::SpHIS5::loxP* | this study |
| HH20 | BY4741, *dph7∆:: loxP::SpHIS5::loxP* | this study |
| HH23 | BY4741, *kti13∆:: loxP::SpHIS5::loxP* | this study |
| HH27 | BY4741, *dph1∆::kanMX4 eft2∆:: loxP::SpHIS5::loxP* | this study |
| HH29 | BY4741, *dph2∆::kanMX4 eft2∆:: loxP::SpHIS5::loxP* | this study |
| HH30 | BY4741, *dph3∆:: loxP::SpHIS5::loxP eft2∆:: loxP::KlURA3::loxP* | this study |
| HH31 | BY4741, *dph4∆::kanMX4 eft2∆:: loxP::SpHIS5::loxP* | this study |
| HH32 | BY4741, *dph5∆::kanMX4 eft2∆:: loxP::SpHIS5::loxP* | this study |
| HH33 | BY4741, *dph6∆::kanMX4 eft2∆:: loxP::SpHIS5::loxP* | this study |
| HH34 | BY4741, *dph7∆::kanMX4 eft2∆:: loxP::SpHIS5::loxP* | this study |
| HH35 | BY4741, *kti13∆::kanMX4 eft2∆:: loxP::SpHIS5::loxP* | this study |
| SUY37 | BY4741, *DPH6-TAP::HisMX6 DPH5-HA::KanMX6* | [2] |

1. Bär C, Zabel R, Liu S, Stark MJ, Schaffrath R (2008) A versatile partner of eukaryotic protein complexes that is involved in multiple biological processes: Kti11/Dph3. Mol Microbiol 69: 1221–1233.

2. Uthman S, Bär C, Scheidt V, Liu S, ten Have S, Giorgini F, et al. (2013) The amidation step of diphthamide biosynthesis in yeast requires *DPH6*, a gene identified through mining the *DPH1-DPH5* interaction network. PLoS Genet 9: e1003334.
