# Supplementary material for: Importance of diphthamide modified EF2 for translational accuracy and competitive cell growth in yeast
Source: PLoS One. 2018 Oct 18;13(10):e0205870. doi: 10.1371/journal.pone.0205870 (PMC6193676; doi:10.1371/journal.pone.0205870)
Supplement: S2 Table — (DOCX) [file pone.0205870.s006.docx]

**S2 Table. Primers used in this study.**

| Primer | Sequence (5’-3’) | Application |
| --- | --- | --- |
| EFT2KOF | TAGCATTCAGACTCAAAGACCACAAACACAAACTATAACATAATTGCAAGCAGCTGAAGCTTCGTACGC | *EFT2* ko |
| EFT2KOR | TTACGACAAAAACTGAAAAAGTTAAATAATTAAAAATTGTTTAACCATTCGCATAGGCCACTAGTGGATCTG | *EFT2* ko |
| EFT2F | GCTGTTGTCCTCCAGGTTATG | *EFT2* ve |
| EFT2R | ACGCGAATAGGCAAAGCAAG | *EFT2* ve |
| DPH1KOF | GCTGCGAATTTTAAGGATAATCGGATAGCTTGAAGCATTTCTTTTTCGTACAGCTGAAGCTTCGTACGC | *DPH1* ko |
| DPH1KOR | TATTTATACATATGTAACAGGAAGACAAGTGACAACAAAAACTATTTAAAGCATAGGCCACTAGTGGATCTG | *DPH1* ko |
| DPH1F | CCATCAGTTTCGACCTCTTG | *DPH1* ve |
| DPH1R | TAGTCACCGGTTGGGCATAG | *DPH1* ve |
| DPH2KOF | CGGGAAGAAATGCGGCAAACGGATCTAAACATGGCAAGGAAGGTGCAGCTGAAGCTTCGTACGC | *DPH2* ko |
| DPH2KOR | CGGCCTTCCACGCGGTCACTTCGCACGGAATGTATATCCCGCATAGGCCACTAGTGGATCTG | *DPH2* ko |
| DPH2F | GGAACGGCCCATTATAGTGC | *DPH2* ve |
| DPH2R | AAACCACACCAGCACAAAGC | *DPH2* ve |
| DPH3KOF | CATACCACGACTGTAAGCACATCATTTGTACAATACATTACCAGCTGCAGCTGAAGCTTCGTACGC | *DPH3* ko |
| DPH3KOR | GCTCTTTCTTTATTTCTATTTGTATTCTCGATCTAGCCTCTCGCATAGGCCACTAGTGGATCTG | *DPH3* ko |
| DPH3F | GCTCTGCTCGCAGCTCAGGATTC | *DPH3* ve |
| DPH3R | CGCCTTTCCGGTGAAATTTG | *DPH3* ve |
| DPH4KOF | TTTATCTCCAATTTAATCTTTCTTTTGGTGTGAAAATTTAGCGAACAGCTGAAGCTTCGTACGC | *DPH4* ko |
| DPH4KOR | ATAAACAGATTTATCTGATATGCTCAATTTCCCCTCCCATTTTCAGCATAGGCCACTAGTGGATCTG | *DPH4* ko |
| DPH4F | GAGCCTACCGATTGGGAATG | *DPH4* ve |
| DPH4R | CTGGCAACGCACCGCGAATAA | *DPH4* ve |
| DPH5KOF | ATGCTTTATTTGATCGGACTTGGTCTCTCGTACAAATCAGACATTACCGTCGACGGCCAGTGAATTCCCGG | *DPH5* ko |
| DPH5KOR | ATAAAAAAGAAACTACACATGAGCGTGTGCATTACCTTTACTCGTCGCTGAGCTTGGCTGCAGGTCGACGG | *DPH5* ko |
| DPH5F | GAGGAGTTGGCTTTCTTCAG | *DPH5* ve |
| DPH5R | AGCGGACATTGCCGTGTACC | *DPH5* ve |
| DPH6KOF | GATAGTTTAATGGTCAGAATGGGCGCTTGTCGCGTGCCAGATCGGGCAGCTGAAGCTTCGTACGC | *DPH6* ko |
| DPH6KOR | CATATTTGGGATCTGTCTATCATTTACACCGCCAGGAGCGTGGACAGCGCATAGGCCACTAGTGGATCTG | *DPH6* ko |
| DPH6F | GGAGAAAGCGCAGAACATC | *DPH6* ve |
| DPH6R | GGGCAAAGCTTAACTTGGTC | *DPH6* ve |
| DPH7KOF | GGGTATTTCTACATCCACCTCTAGCTGGTTTTTGCATAGCTATACAGCTGAAGCTTCGTACGC | *DPH7* ko |
| DPH7KOR | GCCTATATATTAGCCTATATATTAGTCCATATATTGCAGACTGGCATAGGCCACTAGTGGATCTG | *DPH7* ko |
| DPH7F | GCATCCTTCAGCAGGTAACG | *DPH7* ve |
| DPH7R | CAGGAGCCAGCAGGGAGTTTGTTG | *DPH7* ve |
| KTI13KOF | CGCAAGTGATGGAATGTGATCATTAAAGGCTATAACAGGCTTGTATCCAGCTGAAGCTTCGTACGC | *KTI13* ko |
| KTI13KOR | ATGGACATCTATGTATATGATAGTGGGTATATAGTTACTTATCAGGCATAGGCCACTAGTGGATCTG | *KTI13* ko |
| KTI13F | AGTCGGGTGTCCACCAGTAG | *KTI13* ve |
| KTI13R | AGGGATAACGTCAGTCGGAGTTC | *KTI13* ve |

Abbreviations: knock-out (ko), ko verification (ve)
